# Advanced Unsupervised Classification Methods to Detect Anomalies on Earthen Levees Using Polarimetric SAR Imagery

**DOI:** 10.3390/s16060898

**Published:** 2016-06-16

**Authors:** Ramakalavathi Marapareddy, James V. Aanstoos, Nicolas H. Younan

**Affiliations:** 1 Center for Advanced Vehicular Systems, Mississippi State University, Mississippi State, MS 39759, USA; 2 Geosystems Research Institute, Mississippi State University, Mississippi State, MS 39759, USA; aanstoos@gri.msstate.edu; 3 Department of Electrical and Computer Engineering, Mississippi State University, Mississippi State, MS 39762, USA; younan@ece.msstate.edu

**Keywords:** classification, earthen levees, radar polarimetry, Synthetic Aperture Radar, UAVSAR

## Abstract

Fully polarimetric Synthetic Aperture Radar (polSAR) data analysis has wide applications for terrain and ground cover classification. The dynamics of surface and subsurface water events can lead to slope instability resulting in slough slides on earthen levees. Early detection of these anomalies by a remote sensing approach could save time *versus* direct assessment. We used L-band Synthetic Aperture Radar (SAR) to screen levees for anomalies. SAR technology, due to its high spatial resolution and soil penetration capability, is a good choice for identifying problematic areas on earthen levees. Using the parameters entropy (H), anisotropy (A), alpha (α), and eigenvalues (λ, λ_1_, λ_2_, and λ_3_), we implemented several unsupervised classification algorithms for the identification of anomalies on the levee. The classification techniques applied are H/α, H/A, A/α, Wishart H/α, Wishart H/A/α, and H/α/λ classification algorithms. In this work, the effectiveness of the algorithms was demonstrated using quad-polarimetric L-band SAR imagery from the NASA Jet Propulsion Laboratory’s (JPL’s) Uninhabited Aerial Vehicle Synthetic Aperture Radar (UAVSAR). The study area is a section of the lower Mississippi River valley in the Southern USA, where earthen flood control levees are maintained by the US Army Corps of Engineers.

## 1. Introduction

Earthen levees protect large areas of populated and cultivated land in the United States from flooding. The potential loss of life and property associated with the catastrophic failure of levees can be extremely large. Over the entire US, there are more than 150,000 km of levee structures of varying designs and conditions. One type of problem along these levees, which can contribute to failure during a high water event, is the occurrence of slough slides [1]. Slough (or slump) slides are slope failures along a levee, which leave areas of the levee vulnerable to seepage and failure during high water events [2]. The roughness and related textural characteristics of the soil in a slide area affect the amount and pattern of radar backscatter. The type of vegetation that grows in a slide area differs from the surrounding levee vegetation, which can also be used in detecting slides [3].

Polarimetric Synthetic Aperture Radar (PolSAR) data encompass information on scattering mechanisms by diverse target structures and materials. We used multi-polarized L-band Synthetic Aperture Radar (SAR) to screen earthen levees for anomalies. The dynamics of surface and subsurface water events can lead to slope instability resulting in slough slides [4]. If these levees are not healthy, they may not be able to withstand flood conditions which could lead to catastrophic failures. Improved knowledge of the condition of these levees would significantly improve the allocation of precious resources to inspect, test, and repair the ones most in need [5]. Early detection of these anomalies by a remote sensing approach could save time *versus* direct assessment. SAR technology, due to its high spatial resolution and soil penetration capability, is a good choice for identifying problematic areas on levees for this purpose [5]. SAR polarimetry using quad-polarization data is the HV-polarization base in which an antenna transmits and receives horizontally and vertically polarized signals [6].

H/A/α decomposition is an eigenvalue-based decomposition of the coherency matrix 〈$\left[T_{3}\right]$〉 [7,8]. Three features are defined as a function of the eigenvalues and the eigenvectors of 〈$\left[T_{3}\right]$〉: (1) entropy (H), which determines the randomness of scattering or degree of statistical disorder of target; (2) anisotropy (A), which is a unique function of eigenvalue ratios; (3) mean alpha angle (*α*) for different scattering processes and identifying the dominant scattering mechanism [9,10]; and lambda (λ) defined as nonnegative real eigenvalues of the diagonal matrix [Σ_3_] [7,8]. Cloude and Pottier [9] demonstrated an unsupervised classification based on the H/α parameters. These parameters alone were not sufficient for good interclass resolution, indicating that additional information is needed. Hellmann *et al.* [11] tested an unsupervised classification based on the H/α/λ_1_ parameters. However, λ_1_ alone was not able to represent the complete scattering mechanism about the target. Lee *et al.* [12] proposed an unsupervised approach using H and α to initially classify a SAR image and use this classification as training data for a final supervised classification using the maximum likelihood algorithm base on a Wishart distribution. Further improvements can also be made by using the anisotropy parameter. The value of the anisotropy gives the relative significance of secondary scattering mechanisms.

## 2. Method

**Figure 1:**
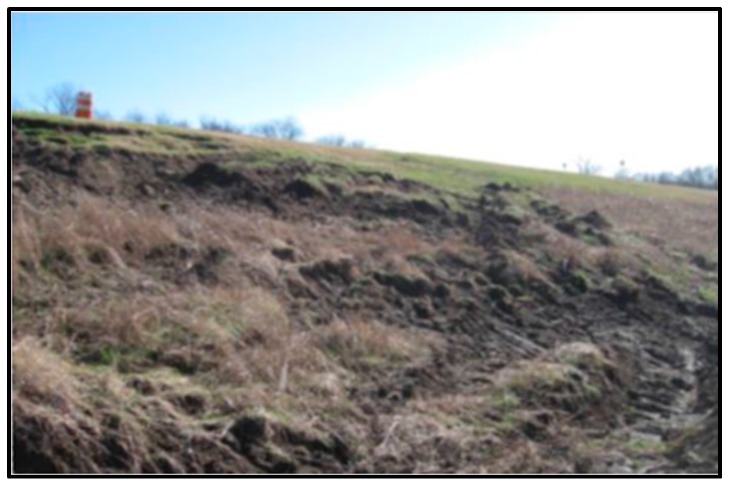
Slough or slump slide on a levee.

**Figure 2:**
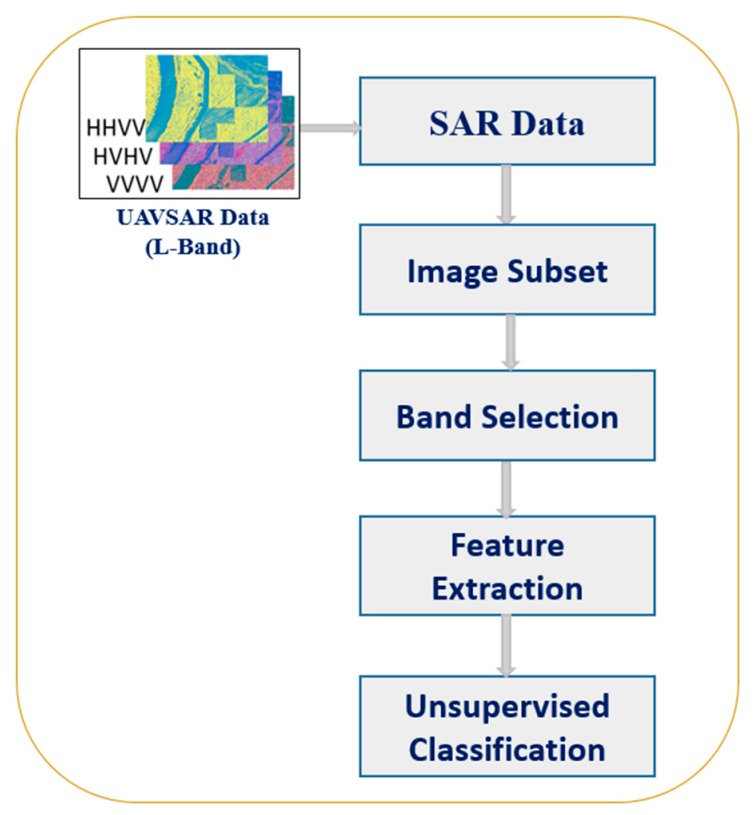
Processing steps for slide detection on levee.

The overall method consists of creating an image subset of the test area, testing our candidate classifiers on the area of interest, and comparing the results to ground truth data. In this paper, we implemented several unsupervised classification algorithms for the identification of anomalies such as slough slides on the levee, an example of which is shown in Figure 1. The classification techniques applied, using H, A, α*,* and λ parameters are H/*α*, H/A, and A/α classification [9,10], Wishart H/α classification [13], Wishart H/A/α classification [14], and extended H/α (*i.e.*, H/α/λ) classification [11,14,15], including classification for individual λ values as H/α/λ_1_, H/α/λ_2_, and H/α/λ_3_. The H/α two dimensional classification employs a three-level Bernoulli statistical model to generate estimates of the average target scattering matrix parameters from the data [9]. The work outlined here is also focused on using λ_1_, λ_2_, and λ_3_, which takes advantage of individual classification using λ_1_, λ_2_, and λ_3_ for a good interclass resolution. In the H/α/λ approach, the backscatter intensity information contained in the eigenvalues λ_1_, λ_2_, and λ_3_ is used to improve the interclass resolution due to the different reflectivities of different scatterers. The classification is performed using the complex data of the Multi-Look Cross products (MLC) acquired by UAVSAR. The MLC data is derived from an average of 3 pixels in range and 12 pixels in azimuth of the single-look complex data (SLC) pixel [13,14,15]. Three complex data bands HHHV, HHVV, and HVVV back scatter magnitudes are used as features for the classification. These processing steps for levee slide detection are illustrated in Figure 2.

### 2.1. Data and Study Area

**Figure 3:**
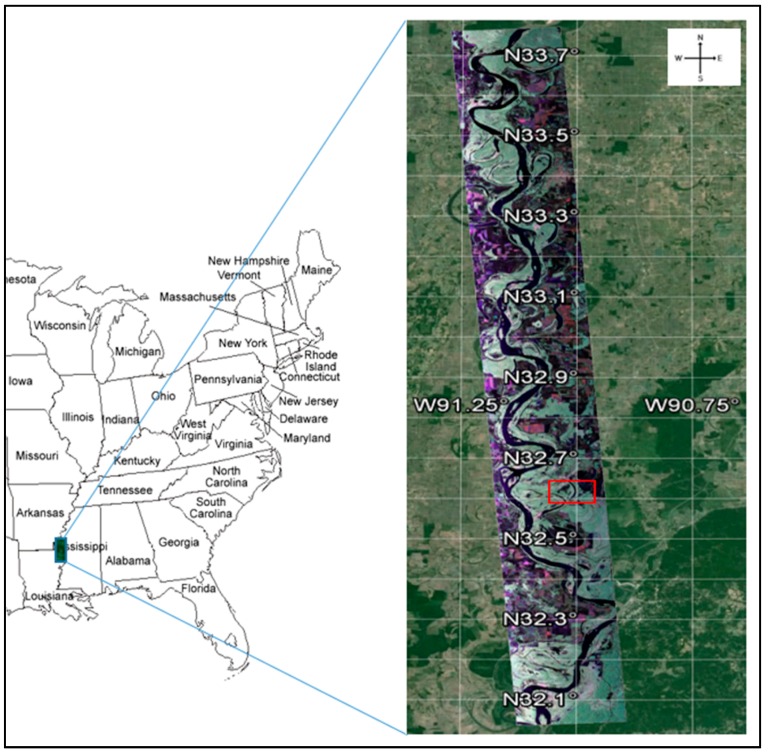
Study area with radar color composite 3 band (HH, VV, & HV) image overlaid on base map.

The study area for this work focuses on the mainline levee system of the Mississippi River along the eastern side of the river in the state of Mississippi [16]. The fully quad-polarimetric L-band (λ = 23.98 cm) SAR imagery from the NASA JPL’s UAVSAR with a range bandwidth of 80 MHz (resulting in better than 2-m range resolution) was used to detect anomalies on earthen levees. The MLC data consists of three sets of complex floating points numbers, 8 bytes per pixel. These complex products are derived from an average of 3 pixels in range and 12 pixels in azimuth, *i.e.*, the number of range and number of azimuth looks are 3 × 12 of the product of each single-look complex data (SLC) pixel, which correspond to HHHV, HHVV, and HVVV. Although the raw ground sample distance is 1.6 by 0.6 m, the multi-look 5 × 7 m data is used to minimize speckle effects [5]. UAVSAR is capable of penetrating dry soil to a few centimeters depth and identifying vertical. Thus, it is valuable in detecting changes in levees that can be used as inputs to a levee monitoring system [16]. We also relied on the ground truth data collected by the US Army Corps of Engineers (USACE) which documented the location and timing of slough slide appearance and repair history. The ground truth data was also compared to optical NAIP (National Agriculture Imagery Program) imagery to visually confirm the slide events. The proposed algorithms were applied to a subset area of a levee. For the multi-polarized SAR imagery, it is useful to create a color composite image from the HH, HV, and VV channels that are being mapped to red, green, and blue, as shown in Figure 3, which includes an overview image overlaid on the base map. This image, collected in a single UAVSAR flight segment flying on a near north heading with the radar looking to the right, has a swath width of 20 km and a total length of 200 km. Across the swath, the beam’s angle of incidence varied from 20 to 65 degrees. It was collected on 25 January 2010. The study area subset is shown in Figure 3 as a red box on the radar image.

### 2.2. Ground Truth Data

The availability of “ground truth” data is a challenge since the targets of interest are portions of levees that show signs of impending failure. Once these are detected, they are quickly repaired depending on their severity [5]. The study area is one in which the levees are managed by the US Army Corps of Engineers and are well-monitored. The Corps, in association with the local levee boards, maintains a good cumulative history of past problems and has particularly identified problematic sections of levees in the study area as shown in Table 1. In addition to the ground truth data provided by the Corps, we conducted field trips at the time of image acquisition to visually inspect the slide area and levee condition. The active slide (Slide 3) was present and unrepaired during the radar image acquisition time on 25 January 2010. Though the date of slide appearance was not identified by the Corps for Slide 3, it is visible in the NAIP (National Agriculture Imagery Program) imagery collected in 2009 and 2010 and was not repaired until after the image acquisition shown in Table 1. Hence, it was an active slide during the time of the image.

### 2.3. The H/A/α/λ Polarimetric Decomposition 

Since the multi-look samples represent spatially average values, the expected value of the 3 × 3 coherency matrix [*T*] is used to represent the averaged distributed target, as in [17]:

(1) $$\langle [T]\rangle =\frac{1}{N}\overset{N}{\underset{i=1}{\sum }}{\underline{k}}_{i}.{\underline{k}}_{i}{}^{*T}=\frac{1}{N}\overset{N}{\underset{i=1}{\sum }}\left[T_{i}\right]\;$$

where the symbol *T stands for complex conjugate.

From this, the 3 × 3 Hermitian coherency matrix, the eigenvectors, and eigenvalues can be used to generate a diagonal form matrix that can be interpreted as the statistical independence among a set of target vectors. The coherency matrix 〈[T]〉 can then be written in this form:

(2) $$\langle [T]\rangle =\left[U_{3}\right]\left[\Sigma \right]{\left[U_{3}\right]}^{-1}\;$$

where [Σ] is a 3 × 3 diagonal matrix with nonnegative real elements (eigenvalues) of 〈[T]〉 and $\left[U_{3}\right]=\left[u_{1}\;u_{2}\;\;u_{3}\right]$ is a 3 × 3 unitary matrix, where $\;u_{1}$,$\;\;u_{2\;}$, and $\;u_{3}$ are the three unit orthogonal eigenvectors of 〈[T]〉, and

(3) $$\left[\Sigma_{3}\right]=\left[\begin{array}{ccc} \lambda_{1} & 0 & 0\\ 0 & \lambda_{2} & 0\\ 0 & 0 & \lambda_{3} \end{array}\right]\;$$

where $\lambda_{1}>\lambda_{2}>\lambda_{3}>0$.

The polarimetric parameterization of the unit target vector *u* involves the combination of three simple scattering mechanisms: surface scattering, double-bounce scattering, and volume scattering, in the case of a distributed target (natural media). These can be characterized from the three components (target generators) of the unit target vector [5,7]. For the mono-static radar case, the 3 × 3 coherency matrix [*T*] has the following parameterization [7]:

(4) $$\left[T\right]=\;\underline{k}.{\underline{k}}^{*T}=\left[\begin{array}{ccc} 2A_{0} & C-jD & H+jG\\ C+jD & B_{0}+B & E+jF\\ H-jG & E-jF & B_{0}-B \end{array}\right]\;$$

Surface Scattering: $A_{0}\gg B_{0}+B,\;B_{0}-B$;

Double-bounce Scattering: $B_{0}+B\;\gg A_{0},\;B_{0}-B$;

Volume Scattering: $B_{0}-B\;\gg A_{0},\;B_{0}+B$.

The Cloude and Pottier [8] decomposition, based on the eigenvalue analysis of a coherency matrix, 〈[T]〉 is

(5) $$\langle [T]\rangle =\;\lambda_{1}u_{1\;}e_{1\;}{}^{*T}+\lambda_{2}u_{2\;}e_{2\;}{}^{*T}+\lambda_{3}u_{3\;}e_{3\;}{}^{*T}\;$$

where $\lambda_{i}$ and $u_{i\;}$ for i=1, 2, 3 are eigenvalues and eigenvectors.

The eigenvectors can be written as

(6) $${\underline{u}}_{i}={\left[\cos\alpha_{i\;}\;\sin\alpha_{i\;}\cos\beta_{i}e^{j\delta_{i}}\;\sin\alpha_{i\;}\cos\beta_{i}e^{j\gamma_{i}}\;\right]}^{T}\;$$

Cloude and Pottier defined three parameters as a function of the eigenvalues and the eigenvectors of 〈[T]〉 [5,7,8,9]: entropy, span(λ), and average alpha angle.

The entropy *H* determines the degree of statistical disorder of each target:

(7) $$H=-\overset{3}{\underset{i=1}{\sum }}P_{i}\log_{3}\left(P_{i}\right)\;,\text{ and}$$

(8) $$P_{i}=\;\frac{\lambda_{i}}{\sum_{j=1}^{3}\lambda_{j}}\;$$

where$\;P_{i}$ is the probability of the eigenvalue$\;\lambda_{i}$.

The entropy, having values in the range 0 to 1, reveals the randomness of the scattering medium, ranging from pure isotropic scattering (*H* = 0) to completely random scattering (*H* = 1). For oceans and low roughness surfaces, *H* is near 0, indicating the dominant mechanism is surface scattering. High values of *H* mean multiple scattering is occurring, as in heavily vegetated areas.

Span (λ) represents the total scattered power:

(9) $$span={\left|S_{HH}\right|}^{2}+{\left|S_{VV}\right|}^{2}\;+2{\left|S_{HV}\right|}^{2}=\overset{3}{\underset{i=1}{\sum }}\lambda_{i}\;$$

(10) $$span=Trace\;\left(\left[\Sigma_{3}\right]\right)\;=\overset{3}{\underset{i=1}{\sum }}\lambda_{i}\;,\text{ and}$$

(11) $$\lambda =\overset{3}{\underset{i=1}{\sum }}\lambda_{i}=\lambda_{1}+\lambda_{2}+\lambda_{3}\;$$

Average alpha angle (α) identifies the dominant scattering mechanism for different scattering processes:

(12) $$\alpha =\;\overset{3}{\underset{i=1}{\sum }}P_{i}\alpha_{i\;}=\;P_{1}\alpha_{1}+P_{2}\alpha_{2}+P_{3}\alpha_{3}\;$$

α reveals the averaged scattering mechanisms from surface scattering (α = 0) to double bounce (α = 90).

### 2.4. Unsupervised H/α Classification

**Figure 4:**
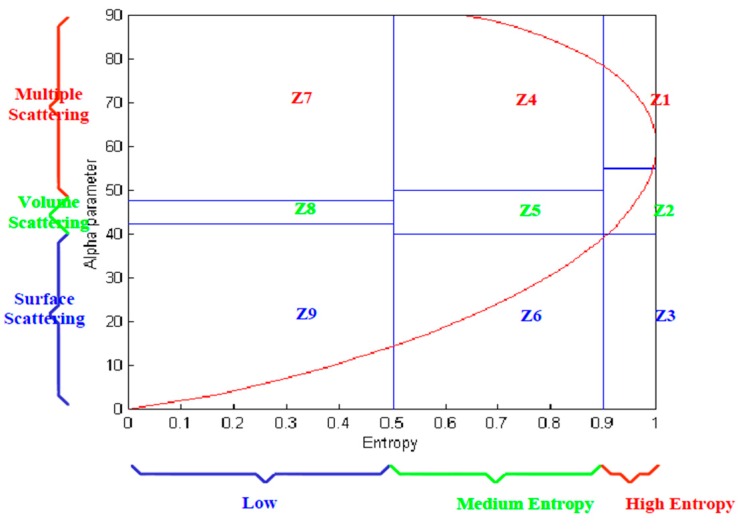
Segmentation of the H/α space [19].

Unsupervised classification schemes were implemented using H and α. All random scattering process can be represented in this 2-dimensional feature space. The underlying principle is that entropy is an indicator of the reversibility of the scattering, while the angle (α) is related to the average scattering mechanism present [17]. The classification plane is divided into nine different zones that represent the different scattering processes, as shown in Figure 4. The classification method is implemented by comparing the observed values of H and α to these fixed zone thresholds to identify the scattering mechanism. The value of α segments the plane into regions characteristic of surface, volume, or multiple scattering. The H or entropy value separates regions of low, medium, and high amounts of randomness along the *x* axis [9,17,18]. The corresponding net combined physical scattering characteristics of each of the zones thus become as follows [9]: Z9: Low Entropy Surface Scattering; Z8: Low Entropy Dipole Scattering; Z7: Low Entropy Multiple Scattering; Z6: Medium Entropy Surface Scattering; Z5: Medium Entropy Vegetation Scattering; Z4: Medium Entropy Multiple Scattering; Z3: (Not a Feasible Region); Z2: High Entropy Vegetation Scattering; and Z1: High Entropy Multiple Scattering.

A refinement of this simple classification was applied next using the method of Lee *et al.* [12]. The initial classification map from the fixed-zone H/α plane defines training sets for a maximum likelihood classification based on the assumption that the data follow a Wishart distribution and the use of the coherency matrix elements as the features. The class of each resulting cluster is determined from whichever zone of the H/α plane the new class center falls in. The classified results are then used as training sets for the next iteration, repeating until a threshold for the percentage of pixels switching classes or an iteration count is met.

### 2.5. H/A/α Unsupervised Classification

A further refinement of the Wishart-based H/α segmentation makes use of an anisotropy parameter during the procedure. This parameter indicates the relative significance of secondary scattering processes. It allows the discrimination of scattering mechanisms having similar entropy values but different eigenvalue distributions. In these cases, greater anisotropy indicates the presence of two dominant scattering processes having equal probability and a less important third mechanism, while low anisotropy reveals a dominant primary scattering process and two secondary mechanisms that are not negligible and have equal significance [17,18,19]. Polarimetric data is first segmented using the maximum likelihood Wishart method. After this procedure is complete, the 8 resulting segments are refined into 16 based on the anisotropy value of each pixel, using a fixed threshold of 0.5. The 16 resulting clusters are then used as training for a second Wishart maximum likelihood classification. The use of anisotropy in the segmentation process allows the splitting of large segments into smaller ones that discriminate small differences in a refined manner, grouping pixels together with similar statistics [14,17,18].

### 2.6. H/α/λ Unsupervised Classification

Cloude and Pottier [9] demonstrated an unsupervised classification based on the H/α parameters. These parameters alone were not sufficient for good interclass resolution, indicating that additional information is needed. Even though the H and α values are derived from fully polarimetric data, they do not completely represent all the polarimetric information. Other parameters, including the span or specific correlation coefficients were expected to significantly improve the classification [20]. Hellmann *et al.* [11,15] tested an unsupervised classification based on the H/α/λ_1_ parameters. However, λ_1_ alone was not able to represent the complete scattering mechanism about the target. The H/α/λ classification including classification for individual λ values such as H/α/λ_1_ is performed for good interclass resolution [12,18]. We implemented classification using H/α/λ, H/α/λ_1_, H/α/λ_2_, and H/α/λ_3_. In the H/α/λ approach, the backscatter intensity information contained in the eigenvalues λ_1_, λ_2_, and λ_3_ is used to improve the interclass resolution due to the differing reflectivities of different scatterers.

## 3. Results and Discussion

**Figure 5:**
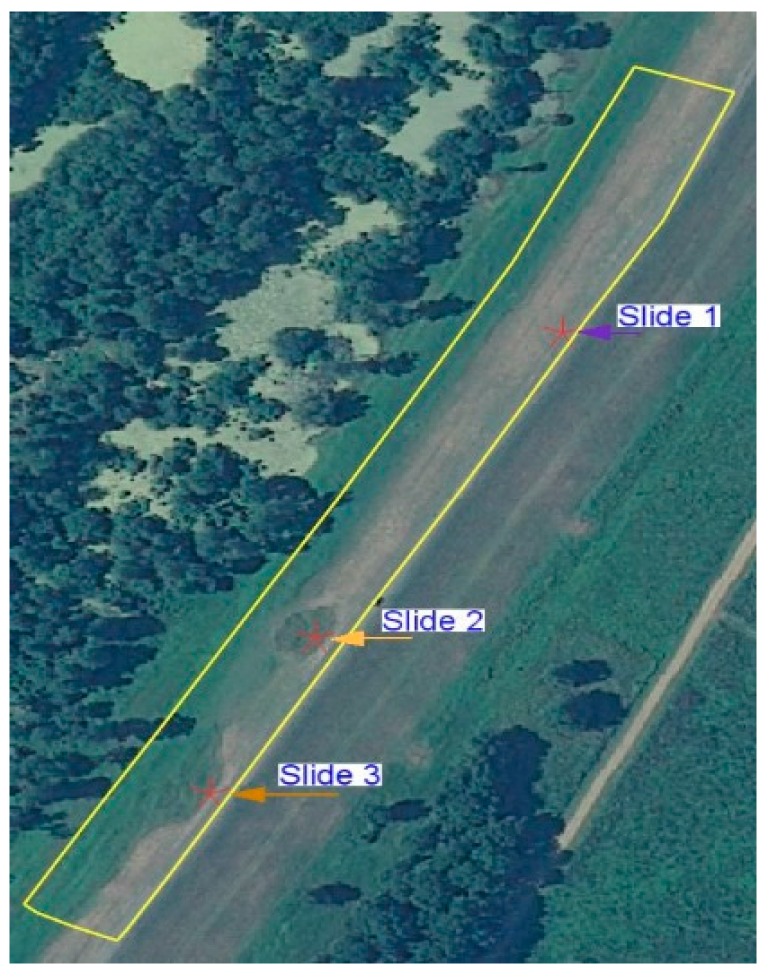
Optical image overlaid with the test area in yellow.

The motivation of this work is to detect slough slides on a levee using remotely sensed imagery. PolSAR data was used for classification of scattering mechanisms of a target, such as surface, double-bounce, or volume scattering [18]. The yellow line overlaid on the optical image in Figure 5 shows the analysis area of the levee from the river side toe to the center line (crown) of the levee. At present, we are focusing on the river side because the chance of the occurrence of slough sides is significantly greater there compared to the land side of the levee. On this image, the locations of the three slides are also indicated.

**Figure 6:**
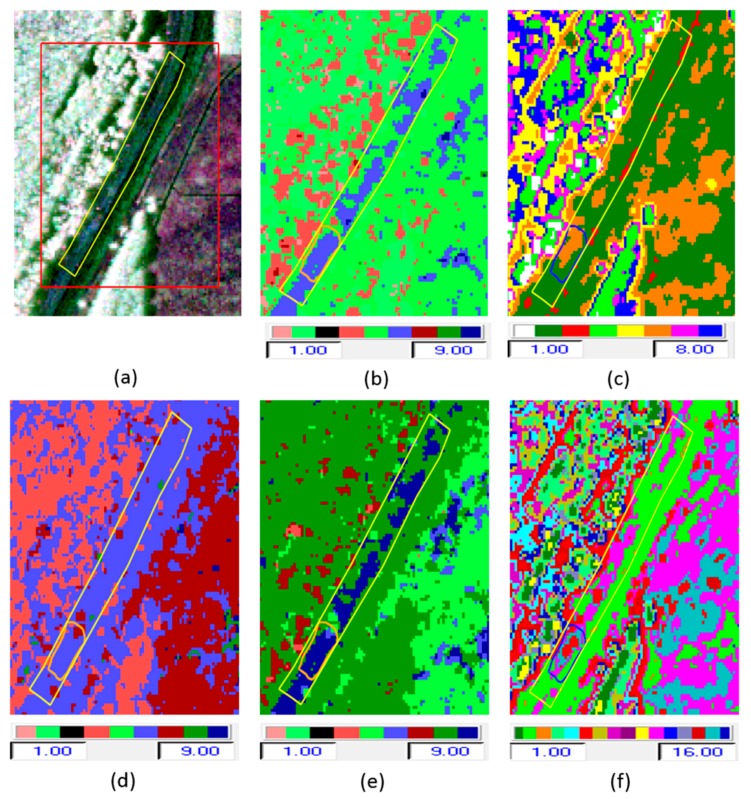
(**a**–**c**) Pauli RGB Image, H/α classification, and Wishart-H/α classification; (**d**–**f**) H/A classification, A/α classification, and Wishart-H/A/α classification.

**Figure 7:**
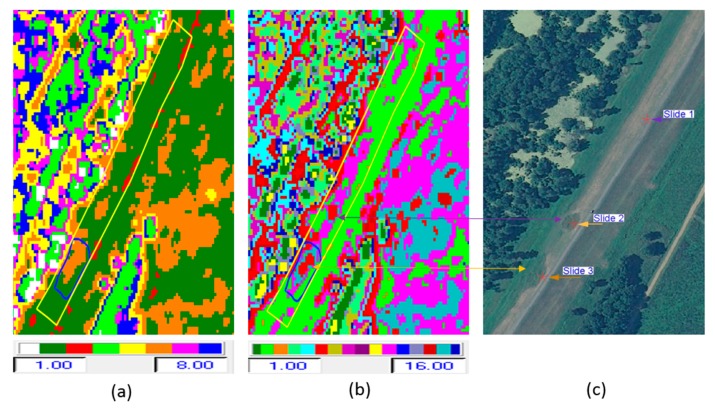
(**a**–**c**) Wishart-H/α classification, Wishart-H/A/α classification, and optical image.

Figure 6a shows the radar backscatter image in the Pauli color composite form (red = HH + VV, green = HH − VV, and blue = HV). In Figure 6b–f, we see respectively the output class map from each of the following classifiers: H/α, Wishart-H/α, H/A, A/α, and Wishart-H/A/α classification. The H/α*,* H/A, A/α*,* and Wishart-H/α classifiers generate nine classes, based on the use of the two-dimensional H/α classification plane. Wishart-H/A/α classification produces 16 classes based on the H/A/α segmentation plane. For the Wishart-H/α and Wishart-H/A/α classification, the polarimetric decomposition parameters (entropy, alpha, and anisotropy) were used as training sets in these iterative classification algorithms. The iteration halted after 10 cycles, when the fraction of class switching was under 10%. Figure 7 repeats the two clearly superior class maps among these—the two Wishart classifier maps—showing how they align with the locations of slides, as shown in the optical image in Figure 7c.

**Figure 8:**
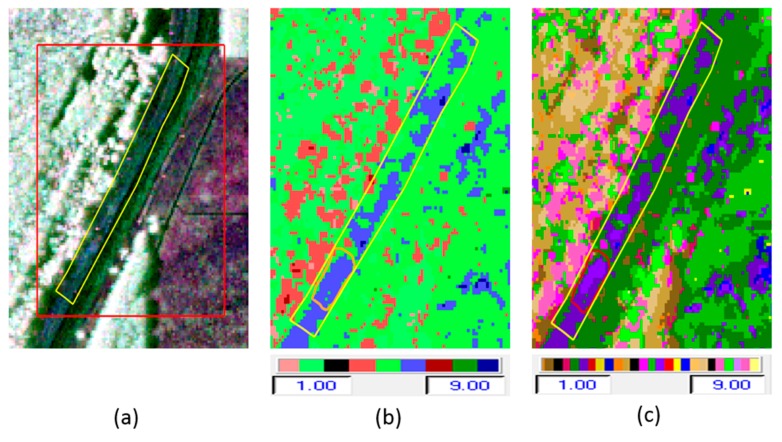

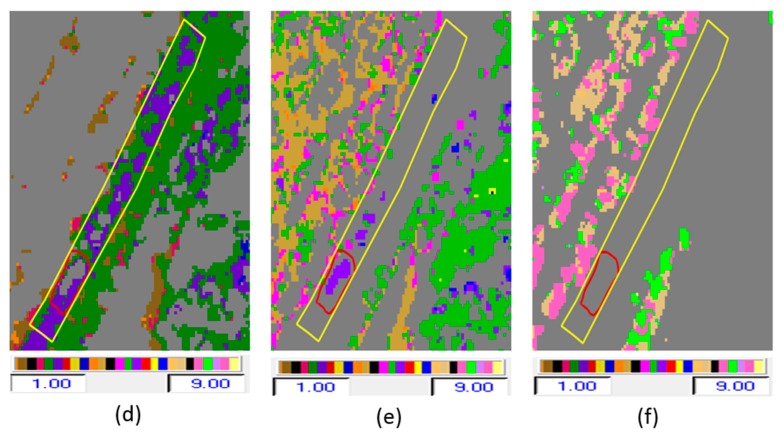
(**a**–**c**) Pauli RGB Image, H/α classification, and H/α/λ classification; (**d**–**f**) H/α/λ_1_ classification, H/α/λ_2_ classification, and H/α/λ_3_ classification.

Figure 8a–f shows: (a) The Pauli RGB image; (b) H/α classification (repeated for comparison); (c) H/α/λ classification; (d) H/α/λ_1_ classification; (e) H/α/λ_2_ classification; and (f) H/α/λ_3_ classification. The latter three classifications are inter-classified within the 9 H/α to represent the interclass resolution due to the different reflectivities of different scatterers [11,15]. All the class values of segmented zones for the H/α, H/α/λ_1_, H/α/λ_2_, and H/α/λ_3_ classifications using the H/α segmentation plane for random media scattering are listed in Table 2. Using these values, the classification color map representing each class of H/α/λ classification was extended from 9 colors to 27 colors. In the classification with individual eigenvalue analysis, the H/α/λ_1_ classification shows where surface scattering dominates; the H/α/λ_2_ classification highlights areas dominated by double-bounce scattering. In the H/α/λ_3_ classification, the volume scattering is emphasized. For the slough slide areas, it can be seen that the surface scattering is partially dominant, the double-bounce scattering is strongly dominant, and the volume scattering is almost zero, since here our target (levee) is naturally distributed.

**Figure 9:**
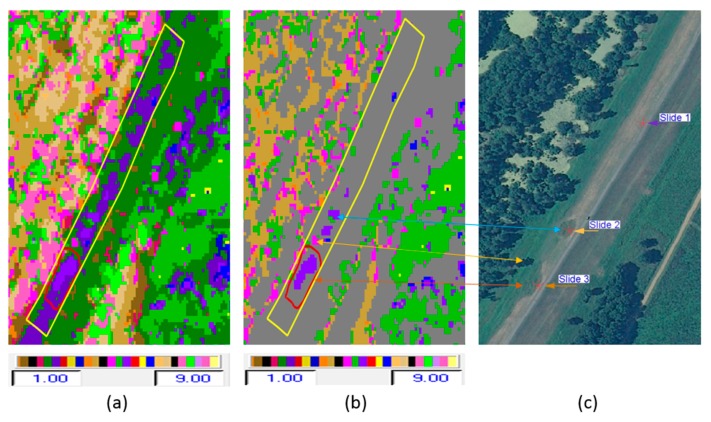
(**a**–**c**) H/α/λ classification, H/α/λ_2_ classification, and optical image.

The slough slide area is marked with a polygon (found in the southern end of the test area) and the test area (on the river side of the levee) is outlined in yellow in the figures. The locations of the three slides are indicated on the optical image with red stars. For this subset, although some of the slide areas (slides 1 and 2) had been repaired by the time of image acquisition, they still show up as anomalies detected by the classification techniques to some extent as shown in Figure 7a–c and Figure 9a–c. Because these slide areas were repaired only two months prior to the time of image acquisition, they still appear anomalous because of the surface roughness and differences in the grass cover. Generally, the healthy levee area has a uniform pattern, but the slide areas have a different pattern in the radar backscattering data [21]. Sometimes other artifacts show similar patterns as the slide area. An example of this is highlighted by the yellow arrow in Figure 9b,c, which is an area influenced by a tall tree nearby casting a radar shadow on the levee. False positives may also occur in the classification process due to rough non-slide surfaces or other anomalies. Specifically, the presence of some anomalous areas in the vicinity of the slide areas may be due to the similarity of soil properties or the vegetation type and condition there, as was verified using *in situ* measurements of soil properties in [22].

A qualitative assessment of the classification results reveals that the Wishart-H/α*,* Wishart-H/A/α*,* and H/α/λ methods provide superior classification for this application compared with the other unsupervised schemes tested. The discrimination of the slide and related anomalies from “healthy” levee areas was effective and was improved by incorporating more parameters. The detected slide and anomalies in the classification results are compared with the optical NAIP image in Figure 7a–c and Figure 9a–c.

**Figure 10:**
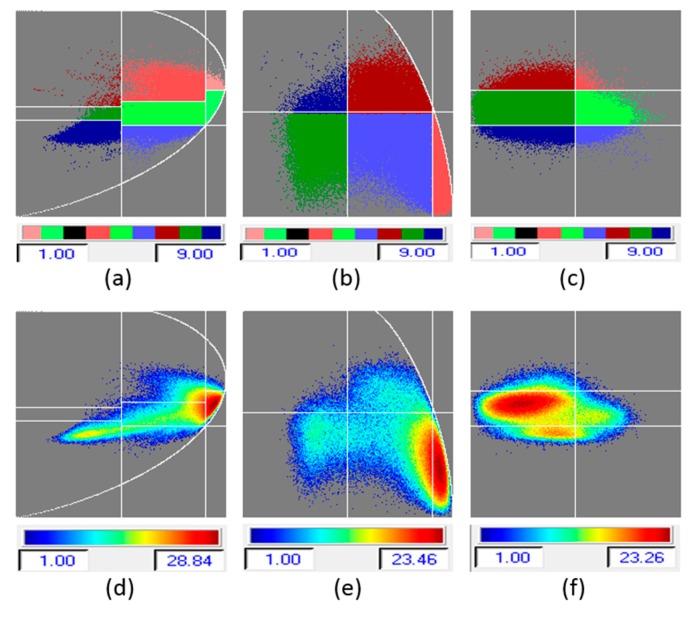
(**a**–**c**) segmented planes for H/α, H/A, and A/α classification; (**d**–**f**) occurrence planes for H/α, H/A, and A/α classification.

The H/α classification uses nine classes, as shown in Figure 6b: out of those, only 4 classes (green, light blue, red, and dark blue) are found in the test area. Other classes occur outside the levee area. This H/α classification does not effectively discriminate the slide area from the non-slide areas. Similarly, the H/A classification and A/α classification result in nine classes as shown in Figure 6d,e, only four of which are found in the test area. The segmented and occurrence planes for the H/α, H/A, and A/α classifications are shown in Figure 10a–f. Once again, the likelihood of identifying slides from this data is not good. This motivated the inclusion of Wishart-based classification, in which the initial classification map defines training sets for classification based on the Wishart distribution iteratively. Significant improvement in each iteration was observed, and the analysis of the final class centers in the two-dimensional H/α classification plane is used for the identification of slides. The Wishart-H/α classification uses eight classes, as shown in Figure 6c, of which for the most part only two classes (green and orange) are found in the test area on the levee. These two classes exhibit good discrimination between slide and nonslide (healthy) areas of the levee.

**Figure 11:**
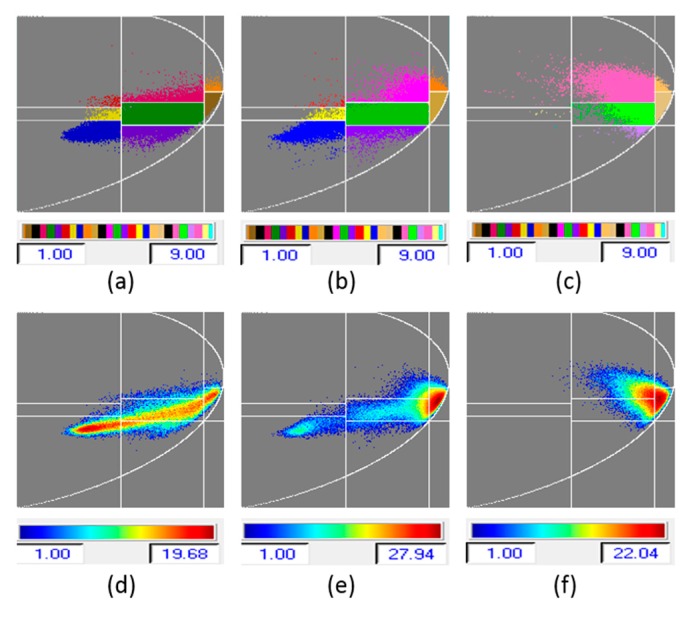
(**a**–**c**) segmented planes for H/α/λ_1_, H/α/λ_2_, and H/α/λ_3_ classification; (**d**–**f**) occurrence planes for H/α/λ_1_, H/α/λ_2_, and H/α/λ_3_ classification.

The Wishart-H/A/α classification is based on 16 classes, as shown in Figure 6f. Out of those, four (colored parrot green, pink, red, and dark ash) occur in the test area. These four classes clearly discriminate the slide and nonslide areas of the levee, as well as distinguishing the area near the slide from other healthy areas on the levee. The segmented and occurrence planes for H/α/λ_1_, H/α/λ_2_, and H/α/λ_3_ classifications are shown in Figure 11a–f. For the slough slide areas, once again it can be seen that the surface scattering is partially dominant, the double-bounce scattering is strongly dominant, and the volume scattering is almost zero. The H/α/λ classification and H/α/λ_2_ classification clearly identified the slide/anomalous areas, as highlighted in Figure 8c,e. The polarimetric SAR data processing and educational tool (PolSARpro v4.2.0 software) from the European Space Agency was used for this work [23].

Although formal quantitative accuracy assessment cannot be performed on unsupervised results without first labeling the output clusters—which is typically done using manual interpretation—we can somewhat quantify the accuracy of this result by noting that a single output cluster dominated the area of the active slide (*i.e.*, Slide 3) and can use it to estimate detection accuracy. In this case, 96% of the slide pixels were detected as such, and as can be seen in Figure 9b, there were very few false positives based on this labeling. Furthermore, almost all of the false positives fall in the area of the recently repaired Slide 2.

## 4. Conclusions

This work presents the results of using SAR data to detect anomalies on an earthen levee. Unsupervised H/α, H/A, A/α, Wishart H/α, Wishart H/A/α*,* and H/α/λ (also individually including λ_1_, λ_2_, and λ_3_) classification algorithms were applied to polarimetric SAR data. The effectiveness of the algorithms is demonstrated using fully quad-polarimetric L-band SAR imagery from the NASA JPL’s UAVSAR. The study area is a section of the lower Mississippi River valley in the Southern USA.

Results show that slough slides on levees exhibit distinctive scattering mechanisms compared with the healthy (*i.e.*, nonslide) areas, and that these differences are revealed by unsupervised classification methods utilizing the polarimetric decomposition parameters H, A, α, and λ. The resulting color-coded class maps can be used to detect anomalous areas on the levee for closer inspection. Wishart-based unsupervised classification schemes clearly show better results for this application. Furthermore, H/α/λ_2_ classification shows noticeably better results to identify slough slide areas. The results indicate that, on the levee, slide areas scatter predominantly as double bounce; meanwhile, in other healthy parts of the levee, surface scattering dominates.

In addition to the active slide area, other anomalous areas are also detected. One interesting point that we noticed is that some of the slide areas that had been repaired just two months prior to the time of image acquisition still appear anomalous because of the texture roughness and differences in grass thickness, and are detected by the classification technique. To validate the attribution of scattering mechanisms such as these to the different surface classes, model-based polSAR decompositions can be used. Early results of that approach were reported in [24].

Although the test study area is small, including only a single active slide area, the methods presented in this paper show promising results. Planned future work includes the use of larger test areas consisting of more active slides, seasonal images acquired by the polSAR, and different geometrical orientations of the levee.

## Figures and Tables

**Table 1:** Updated slides ground truth from Mississippi Levee Board.

Slide Number	Latitude North	Longitude West	Date Slide Appeared	Date Slide Repaired
1	32 36 37.7N	90 59 42.3W	October 2009	November 2009
2	32 36 32.0N	90 59 46.3W	August 2008	November 2009
3	32 36 29.1N	90 59 48.0W	-	September 2010

**Table 2:** Class values of segmented zones for the H/α, H/α/λ_1_, H/α/λ_2_, and H/α/λ_3_ classifications using the H/α segmentation plane for random media scattering.

Zone/Class Value	Classification			
	H/α	H/α/λ_1_	H/α/λ_2_	H/α/λ_3_
Z1	1	1	3.37	6.54
Z2	2	1.31	4.08	6.85
Z3	0	0	0	0
Z4	4	1.92	4.69	8.38
Z5	5	2.23	5.00	7.77
Z6	6	2.54	5.31	8.08
Z7	7	2.85	5.62	8.38
Z8	8	3.15	5.92	8.69
Z9	9	3.46	6.23	0
